# Major pulmonary resection after neoadjuvant chemotherapy or chemoradiation in potentially resectable stage III non-small cell lung carcinoma

**DOI:** 10.1038/s41598-021-99271-3

**Published:** 2021-10-12

**Authors:** Michael Peer, Sharbel Azzam, Arnold Cyjon, Rivka Katsnelson, Henri Hayat, Ilan Bar, Ofer Merimsky

**Affiliations:** 1 Department of Thoracic Surgery, Tel Aviv Medical Center, Affiliated with Sackler School of Medicine, Tel-Aviv University, Tel Aviv, Israel; 2 Department of Oncology, Shamir Medical Center, Zerifin, Affiliated with Sackler School of Medicine, Tel-Aviv University, Tel Aviv, Israel; 3 Department of Oncology, Kaplan Medical Center, Rehovot, Affiliated with the Hebrew University of Jerusalem, Medical School, Jerusalem, Israel; 4 Department of Oncology, Wolfson Medical Center, Holon, Affiliated with Sackler School of Medicine, Tel Aviv University, Tel-Aviv, Israel; 5 Department of Thoracic Surgery, Kaplan Medical Center, Rehovot, Affiliated with the Hebrew University of Jerusalem, Medical School, Jerusalem, Israel; 6 Department of Oncology, Tel Aviv Medical Center, Tel Aviv, Affiliated with Sackler School of Medicine, Tel-Aviv, Israel

**Keywords:** Lung cancer, Non-small-cell lung cancer

## Abstract

The aim of this study was to identify predictors of postoperative outcome and survival of locally advanced non-small cell lung carcinoma (NSCLC) resections after neoadjuvant chemotherapy or chemoradiation. Medical records of all patients with clinical stage III potentially resectable NSCLC initially treated by neoadjuvant chemotherapy or chemoradiation followed by major pulmonary resections were retrieved from the databases of four Israeli Medical Centers between 1999 to 2019. The 124 suitable patients included, 86 males (69.4%) and 38 females (30.6%), with an average age of 64.2 years (range 37–82) and an average hospital stay of 12.6 days (range 5–123). Complete resection was achieved in 92.7% of the patients, while complete pathologic response was achieved in 35.5%. The overall readmission rate was 16.1%. The overall 5-year survival rate was 47.9%. One patient (0.8%) had local recurrence. Postoperative complications were reported in 49.2% of the patients, mainly atrial fibrillation (15.9%) and pneumonia (13.7%), empyema (10.3%), and early bronchopleural fistula (7.3%). The early in-hospital mortality rate was 6.5%, and the 6-month mortality rate was 5.6%. Pre-neoadjuvant bulky mediastinal disease (lymph nodes > 20 mm) (p = 0.034), persistent postoperative N2 disease (p = 0.016), R1 resection (p = 0.027), preoperative N2 multistation disease (p = 0.053) and postoperative stage IIIA (p = 0.001) emerged as negative predictive factors for survival. Our findings demonstrate that neoadjuvant chemotherapy or chemoradiation in locally advanced potentially resectable NSCLC, followed by major pulmonary resection, is a beneficial approach in selected cases.

## Introduction

Stage III non-small cell lung carcinoma (NSCLC) is a very heterogeneous disease that depends upon the tumor size (T1–4), tumor local extension and extension of nodal involvement. N2 disease is heterogeneous by itself, as it may include several options depending on the site, number of stations, and nature of the nodes (i.e., bulky or separate). Stage IIIA disease is usually approached by preoperative induction therapy followed by surgical resection. Postoperative treatment with anti-EGFR osimertinib has been shown to improve disease-free survival in patients with EGFR-mutated tumors^1^. On the other hand, IIIB disease may be managed by chemoradiation followed by immunotherapy (PACIFIC trial)^2^. Selected cases of IIIB disease may be treated by induction therapy followed by curative surgery only. Postoperative immunotherapy in these cases has not yet been defined. The goals of induction therapy are downstaging and downsizing the primary disease to improve the resectability and eradication of systemic micrometastatic disease. Patients whose disease is downstaged may be good candidates for surgery.

Surgery, however, is still associated with an increased incidence of postoperative morbidity and mortality, with pneumonectomy being associated with high complication rates^3,4^.

Skilled surgeons, modern surgical techniques and perioperative care in highly specialized thoracic intensive care units are key for improving postoperative outcomes. Here, we analyze our results in treating stage IIIA/B potentially resectable NSCLC and investigate the factors that have affected patient outcomes.

## Patients and methods

### Patients

We retrospectively reviewed the data of 124 patients with a confirmed diagnosis of stage IIIA/B NSCLC. All patients were oncologically treated and followed in one of four Israeli centers, Tel Aviv Medical Center (Tel Aviv), Shamir Medical Center (Zerifin), Kaplan Medical Center (Rehovot), and Wolfson Medical Center (Holon), from May 1999 to December 2019. Follow-up data were available for 121 patients.

There were 86 (69.4%) males and 38 females (30.6%) with an average age of 64.2 years (range 37–82). One-hundred and ten patients (88.7%) were smokers. The retrieved baseline data comprised patient demographics, comorbidities, induction therapy, primary tumor size, location, histology, side and type of surgery, stages at diagnosis (clinical) and postoperative stages (pathologic), postoperative outcomes, including complications, morbidity, mortality, length of hospital stay, readmission, and local recurrence rates.

### Methods

The initial treatment plan of each case along with medical treatment results and options for surgery or immunotherapy were discussed and approved by a multidisciplinary team at tumor board meetings. In all cases, induction chemotherapy employing a platinum-based chemotherapy regimen was administered, together with a second agent such as paclitaxel, etoposide, vinorelbine or pemetrexed, depending on the tumor histology (checkpoint inhibitors were not registered for induction therapy and were not available for induction protocols).

Radiation therapy was given concurrently (60 Gy/30 courses, 5 days weekly) with chemotherapy in 85 patients [68.5%], starting from cycle one, 2 or 3, depending on the availability of the radiation service. A radiation dose of 60 Gy is used in our centers for induction as a definitive therapy. Thirty-nine patients (31.5%) received chemotherapy only. The time elapsed between the end of the induction therapy and the date of surgery was 4–6 weeks. All patients underwent resection by a permanent thoracic-oncology surgical team.

### Preoperative workup and tumor classification

The diagnostic workup/staging included a complete medical history and physical examination, chest radiography, bronchoscopy, contrast-enhanced computed tomography (CT) of the chest, electrocardiography, and complete blood counts, chemistry profiles, and coagulation tests. All patients underwent pretreatment (neoadjuvant therapy) and posttreatment (preoperative) restaging by positron-emission tomography-CT (PET-CT) or contrast-enhanced CT of the chest, as well as contrast-enhanced CT of the brain.

Congestive heart failure was defined as a reduced ejection fraction of less than 45%. Cardiac comorbidity was defined as the presence of coronary artery disease or any previous cardiac surgery or catheterization, current cardiac failure, or arrhythmia. Chronic renal failure (CRF) was defined as an elevated creatinine level of > 1.5 mg/dl. Chronic obstructive pulmonary disease (COPD) was defined as a forced expiratory volume in 1 s/forced vital capacity ratio less than 70%.

Tumors were classified and staged preoperatively and postoperatively according to the 1997 International System for Staging Lung Cancer^5^. Most of the patients had either squamous cell carcinoma or adenocarcinoma (37.9% and 41.1%, respectively). Pretreatment mediastinal staging was performed by cervical mediastinoscopy (27 patients, 21.8%) or endobronchial ultrasound (EBUS) (16 patients, 12.9%), when enlarged (> 1.0 cm) mediastinal lymph nodes were seen on CT or when high fluorodeoxyglucose (FDG) uptake was seen in mediastinal lymph nodes on PET-CT (performed in 92 patients, 74.2%). Chest wall involvement was classified as invasion of the diaphragm, chest wall muscles, or ribs. Involvement of mediastinal structures was classified as invasion of the mediastinal pleura (or pericardium), great vessels (aorta), esophageal wall, vertebral bodies, trachea, carina, or recurrent laryngeal nerve. Fifteen patients had been diagnosed preoperatively as having superior sulcus (Pancoast) tumors (SSTs) (12.1%).

Single-Station N2 disease was classified when only one mediastinal station lymph node was positive according to PET-CT results (generally, R4, 7 or L5). Multistation N2 disease was classified when lymph node involvement was identified by PET-CT in at least one mediastinal and one or more hilar and mediastinal stations or according to the size of the mediastinal lymph nodes (> 10 mm) on CT of the chest in the pre-PET era. At least three hilar and mediastinal station lymph nodes were routinely dissected or sampled within anatomical landmarks during pulmonary resection.

### Surgical technique

All of the study patients underwent standard anesthesia with a double-lumen endotracheal tube, perioperative low thoracic epidural analgesia, and surgery by means of a similar technique consisting of a standard serratus muscle-sparing posterolateral thoracotomy in the fifth or sixth intercostal space^6^. Pulmonary resections were performed according to the European Society of Thoracic Surgeons Guidelines^7^ within four to six weeks of the completion of induction therapy. Mechanical staples were used for the closing of pulmonary veins, pulmonary arteries, and bronchi. The bronchial stumps were reinforced with viable intercostal, serratus or latissimus muscle flaps in selected cases (5 patients, 4.0%). Additional thoracic structures and/or the mediastinal pleura, including the pericardium, were resected in cases of local invasion. Pericardium reconstruction was performed by using bovine pericardium (Gore-Tex soft tissue patch, Delaware Corp, Newark) in cases of intra-pericardial resections. One 36 French chest tube was placed in the empty chest cavity, and it was generally removed within 24 h after the surgery in cases of pneumonectomy. Two 36 French chest tubes (one of them curved and one straight) were used in cases of bilobectomy or lobectomy.

Generally, patients were extubated in the recovery room, initially monitored in the high-dependency unit for 24–48 h and, thereafter, transferred to the thoracic surgery department intensive care ward. Early hospital mortality was defined as death occurring during the postoperative hospitalization period. Late mortality was defined as death occurring within 6 months after surgery. The patients were followed postoperatively for cancer recurrence and survival every 3 months for the first year and every 6 months thereafter (mean: 43.6 months), and the final data on survival were recorded on January 1, 2020.

### Statistical analysis

Due to the heterogeneity of stage III NSCLC, we evaluated different parameters of patients with locally advanced potentially resectable disease separately and together to analyze the factors that potentially influence postoperative outcome or survival. Such factors included the following:

- postoperative complications: atelectasis, mechanical ventilation, atrial fibrillation, acute renal failure, empyema, tracheostomy, pneumonia, early and late bronchopleural fistula, acute respiratory distress syndrome (ARDS), air leak, intraoperative hemorrhage and recurrent laryngeal nerve palsy;
- sex and comorbidities: coronary artery disease (CAD), chronic obstructive pulmonary disease (COPD), peripheral vascular disease (PVD), chronic renal failure (CRF), hypothyroidism, obesity, noninsulin-dependent diabetes mellitus (NIDDM), hypertension (HTN), peptic disease, and smoking;
- histologic type of tumor, side of surgery, kind of surgery (pneumonectomy, lobectomy, bilobectomy), extent of surgery (extrapleural, intra-pericardial and completion resection), and tumor location (endobronchial, Pancoast tumors, subcarinal and paraesophageal), and local recurrence;
- preoperative treatment (chemotherapy and chemoradiation), pre-neoadjuvant PET-CT, pre-neoadjuvant mediastinal staging (N2 negative or N2 single positive lymph node and multiple positive N2 lymph nodes), pre-neoadjuvant size of mediastinal lymph nodes (< 20 mm and > 20 mm), pretreatment staging (IIIA & IIIB), pathologic postoperative staging (IA–IV and complete pathologic response, CPR), surgical margins (R0 & R1), persistent N2 disease, visceral and parietal pleural and vascular invasion.

Patients and surgical and postoperative characteristics were evaluated; continuous variables with a normal distribution were summarized as means and standard deviations and compared using independent t-tests. Variables that deviated from a normal distribution were summarized as medians and IQRs (interquartile ranges) and compared using the Mann–Whitney test. Categorical variables were summarized as counts and percentages and compared using the chi-square test. The primary endpoint was death.

The cumulative rates of death were compared using Kaplan–Meier curves, and the Cox regression model was applied to evaluate the adjusted effect of surgery and patient characteristics on patient survival. The figures were generated using IBM Corp. Released 2020. IBM SPSS Statistics for Windows, Version 27.0. Armonk, NY: IBM Corp.

A two-sided p value less than 0.05 was considered to define statistical significance. Statistical analyses were carried out using IBM Corp. Released 2020. IBM SPSS Statistics for Windows, Version 27.0. Armonk, NY: IBM Corp.

### Ethical approval

The Institutional Review Board of Shamir Medical Center (formerly Assaf Harofeh), a referral center of thoracic surgery until 2019, approved this retrospective study and waived informed consent.

## Results

All patients underwent major pulmonary resections due to locally advanced potentially resectable stage IIIA/B NSCLC. Patient demographics, comorbidities, histological type of tumor, type and side of surgery, induction treatment, and other characteristics are summarized in Table 1. Pneumonectomy was performed in 61 patients (49.2%, completion pneumonectomy in 8 patients (6.5%), extrapleural pneumonectomy in 8 patients (6.5%), intra-pericardial pneumonectomy in 7 patients (5.6%), bilobectomy in 5 patients (4.0%), lobectomy in 58 patients (56.8%), and extrapleural lobectomy in 14 patients (11.3%). Thirteen patients underwent chest wall resection (10.5%), two patients underwent vertebral body resection (1.6%), and seven patients (5.6%) underwent pericardium resection and reconstruction. The data on the clinical staging of the patients before admission to induction therapy and on the pathologic staging of the 124 patients without radiologic disease progression are summarized in Tables 2 and 3, respectively. The average number of lymph nodes dissected or sampled during each surgery was 8.6 (range 0–18). The data on the hilar and mediastinal station lymph nodes are summarized in Table 4.

**Table 1:** Demographic, comorbid, radiologic, histologic, and surgical characteristics of the 124 patients.

Variable	Number	% (p value)	
Male	86	69.4(0.118)	
Female	38	30.6(0.118)	
**Surgical procedures**			
Right-sided	74	59.7(0.368)	
Left-sided	50	40.3(0.368)	
Pneumonectomy	61 (right = 31, left = 30)	49.2(0.142)	
Intrapericardial	7	5.6 (0.048)	
Extrapleural	8	6.5 (0.242)	
Completion	8	6.5 (0.999)	
Bilobectomy	5 (RUL/RML = 1, RML/RLL = 4)	4.0(0.142)	
Lobectomy	58 (RUL = 32, LUL = 18, RLL = 6, LLL = 2)	46.8(0.142)	
**Histologic type of tumors**			
Adenocarcinoma	51	41.1(0.673)	
Squamous cell carcinoma	47	37.9(0.673)	
Large cell carcinoma	10	8.1	
Poorly differentiated carcinoma	11	8.9	
Other carcinoma	5	4.0	
**Comorbidities**			
Chronic obstructive pulmonary disease	54	43.5(0.511)	
Peripheral vascular disease	15	12.1(0.907)	
Noninsulin-dependent diabetes mellitus	22	17.7(0.194)	
Obesity	23	18.5(0.991)	
Other malignancy	29 (7 = secondary primary lung carcinoma)	23.4	
Coronary artery disease	30	24.2(0.290)	
Hypertension	17	13.7(0.954)	
Peptic disease	4	3.2 (0.347)	
Chronic renal failure	3	2.4 (0.999)	
Cerebrovascular accident	6	4.8 (0.417)	
Hypothyroidism	3	2.4**(0.543)**	
Liver disease	2	1.6	
Drug/alcohol abuse	3	2.4	
Chronic heart failure	5	4.0	
Pulmonary edema	3	2.4	
Chronic atrial fibrillation	3	2.4	
**Pretreatment staging**			
IIIA	91	73.4(0.812)	
IIIB	33	26.6(0.812)	
Smokers	110	88.7(0.556)	
**Type of induction treatment**			
Chemoradiation	85	68.5(0.066)	
Chemotherapy	39	31.5(0.066)	
PET-CT	92	74.2(0.001)	

*RUL* right upper lobe, *RML* right middle lobe, *RLL* right lower lobe, *LUL* left upper lobe, *LLL* left lower lobe.

**Table 2:** Clinical staging of 124 patients with locally advanced stage IIIA/B NSCLC before admission to neoadjuvant chemotherapy or chemoradiation.

cTNM	Involvement of different thoracic structures	Total
**Stage IIIA**		91 (73.4%)
T1N2	N2 single—3, N2 multiple—7	10
T2N2	N2 single—10, N2 multiple—27	37
T3N2	Main bronchus involvement—2 (N2 single—1, N2 multiple—1) Chest wall involvement—5 (N2 single—2, N2 multiple—3)	7
T4N0	(SST, Pancoast tumor)—10, mediastinal structure involvement (mediastinal pleural & pericardium & recurrent laryngeal nerve) & aorta & carina & vertebral body^a^—17, 4, 4, and 2 patients, respectively	37
**Stage IIIB**		33 (26.6%)
T4N2	Two satellite lesions—4 (N2 single—1, N2 multiple—3); mediastinal structures involvement (mediastinal pleural & pericardium & recurrent laryngeal nerve)—10 (N2 single—3, N2 multiple—7); superior sulcus tumors (SST, Pancoast tumor)—4 (N2 single—2, N2 multiple -2); aorta & esophagus & carina involvement—9: 6 (N2 single—3, N2 multiple -3) & 2 (N2 single—1, N2 multiple—1) & 1 (N2 single), respectively	27
T2N3	N3 single—2	2
T3N3 T4N3	Main bronchus involvement -1 (N3 single—1) Mediastinal structure involvement (mediastinal pleural & pericardium)—2 (N3—single) & aorta—1 (N3 single)	1 3
Total^b^		124

cTNM—recommendations for clinical stage groups.

^a^Superior sulcus tumor (SST) —1 patient.

^b^Intrabronchial tumors—7 patients.

**Table 3:** Surgical staging of 124 patients with locally advanced stage IIIA/B NSCLC after neoadjuvant chemotherapy or chemoradiation.

	No residual disease	IA	IB	IIA	IIB	IIIA	IIIB	IV	Total
Total (p value)	44 (35.5%) (p = 0.001)	24 (19.4%) (p = 0.423)	17 (13.7%) (p = 0.229)	8 (6.5%) (p = 0.260)	10 (8.1%) (p = 1.000)	18 (14.5%) (p = 0.001)	1 (0.8%) (p = 0.999)	2 (1.6%) (p = 0.999)	124

**Table 4:** Total number of lymph nodes dissected in 124 patients according to hilar and mediastinal stations.

Lymph node stations	Negative	Positive	Total
R4	153	20	173
7 (rt & lt)	117	10	127
R8	11	7	18
R9	23	3	26
L5	67	9	76
L9	27	2	29
R10	81	15	96
R11 & 12	261	37	298
L10	44	3	47
L11 & 12	109	13	121
Total	893	119	1012

The average hospital stay for the operated patients was 12.6 days (range 5–123). Postoperative complications were sustained by 61 patients (49.2%), including seven patients with intraoperative hemorrhage (5.6%). Atrial fibrillation and pneumonia were the most common postoperative complications (19 (15.9%) and 17 (13.7%) patients, respectively). Lobar atelectasis confirmed by chest radiography and treated by immediate bronchoscopy was recorded for 11 patients (8.9%). A prolonged air leak defined as an air leak documented one week after lobectomy or bilobectomy was recorded for six patients (4.8%). Empyema occurred in 13 patients (10.5%), early broncho-pleural fistulas (BPFs, during the first three postoperative months) occurred in nine patients (7.3%), and late BPF (between 3 and 6 months postoperatively) occurred in two patients (1.6%). In two patients (1.6%), BPF was treated by Amplatzer device implantation (Amplatzer PFO Occluder Corp, USA) through postpneumonectomy and post-bilobectomy stumps^6^. Empyema in postpneumonectomy patients was treated by video-assisted thoracoscopic surgery (VATS) in five patients (4.9%), by three-rib open window thoracoplasty in seven patients (5.5%), and by sternotomy with trans-pericardial BPF repair in one patient (0.8%). Prolonged mechanical ventilation was required postoperatively in 10 patients (8.1%), of whom three developed acute respiratory distress syndrome (ARDS) (2.4%), two developed pulmonary edema (PE) (1.6%), and five required a tracheostomy (4.0%). The overall readmission rate was 16.1% (20 patients).

Early (in-hospital) mortality was recorded for eight patients (6.5%), five after pneumonectomy (three right-sided and two left-sided), one after bilobectomy, (right middle lobe (RML) and right lower lobe (RLL)), and two after lobectomy (right upper lobe (RUL)). The causes of death were ARDS in three patients, pneumonia in two patients, complicated BPF with empyema in two patients, and empyema without BPF in one patient. Late (6 months) mortality was recorded for seven patients (5.6%): four after pneumonectomy (three right-sided and one left-sided), one after bilobectomy (RML/RLL), and two after lobectomy (RUL and left upper lobe (LUL)). The causes of death were empyema without BPF in one patient, complicated BPF with empyema in two patients, metastatic disease in three patients, and pneumonia in one patient. The data relating to all types of postoperative complications are summarized in Table 5.

**Table 5:** Overall complications, mortality, and hospital stay of 124 locally advanced NSCLC patients who underwent major pulmonary resection after induction therapy.

Complications	No. patients	%	p value
Overall complications	61	49.2	
Atelectasis lobar radiologically confirmed	11	8.9	(p = 0.229)
Tracheostomy	5	4.0	(p = 0.162)
Prolonged (> 48 h) mechanical ventilation	10	8.1	(p = 0.999)
Atrial fibrillation	19	15.3	(p = 0.175)
Pneumonia	17	13.7	(p = 0.163)
Hospital/30-day mortality	8	6.5	
Late/6-month mortality	7	5.6	
Intraoperative hemorrhage	7	5.6	(p = 0.420)
Early (< 3 months) bronchopleural fistula	9	7.3	(p = 0.999)
Late (> 3 months) bronchopleural fistula	2	1.6	(p = 0.347)
Empyema	13	10.5	(p = 0.371)
Readmission	20	16.1	
Prolonged > 1 week air leak post lobectomy	6	4.8	(p = 0.664)
Acute respiratory distress syndrome	3	2.4	(p = 0.551)
Chylothorax	1	0.8	
Mediastinal shift	2	1.6	
Vocal cord palsy	3	2.4	(p = 0.658)
Ischemic central & peripheral event	2	1.6	
Pulmonary thromboembolism	2	1.6	
Pulmonary edema	2	1.6	
Acute renal failure	2	1.6	(p = 0.543)
Delirium	1	0.8	

A complete pathologic response (CPR) (i.e., no residual tumor with free lymph nodes) was recorded in 44 patients (35.5%), and microscopic residual tumor (< 10 mm) was identified in 13 patients (10.5%). R0 resection (i.e., complete resection of the tumor with free margins and negative highest mediastinal lymph nodes) was achieved in 115 patients (92.7%), and local recurrence was seen in one patient (0.8%).

Most of the factors examined in the study were found to be nonsignificant (Tables 1, 2, 3, 4).

**Figure 1:**
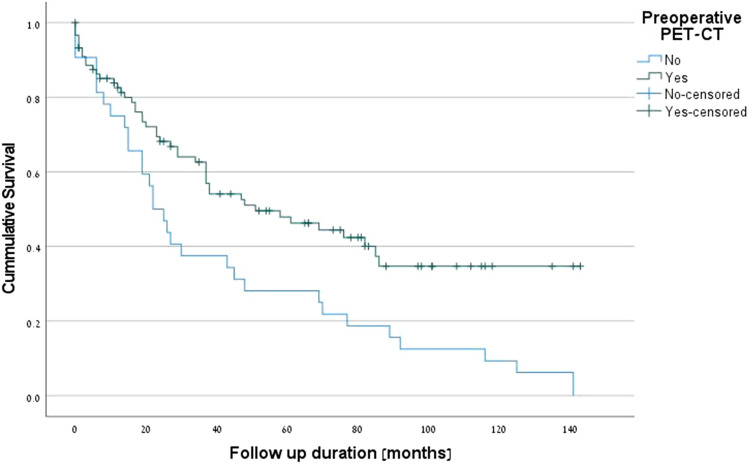
Kaplan–Meier survival curves for preoperative PET-CT.

**Figure 2:**
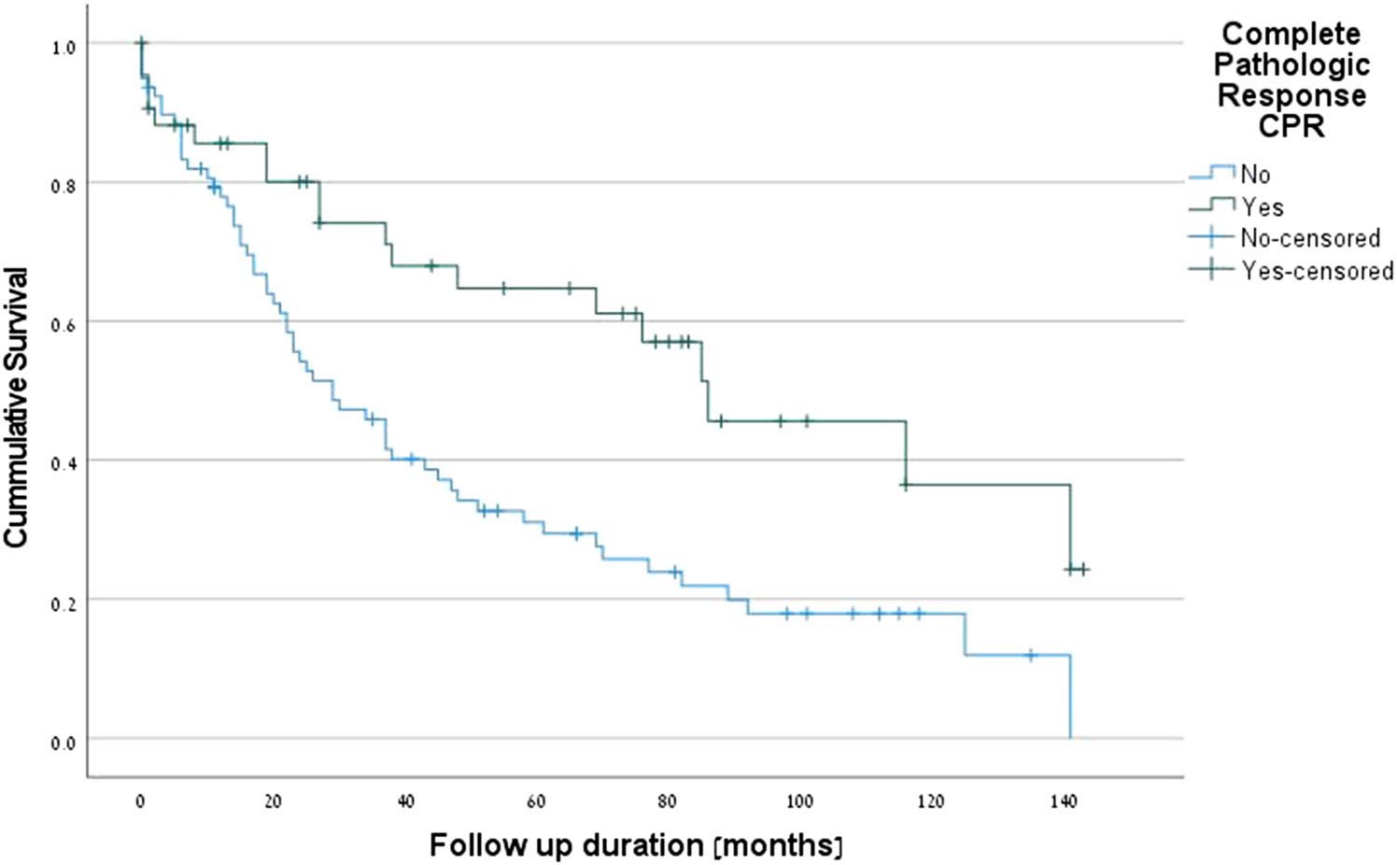
Kaplan–Meier survival curves for complete pathologic response (CPR).

**Figure 3:**
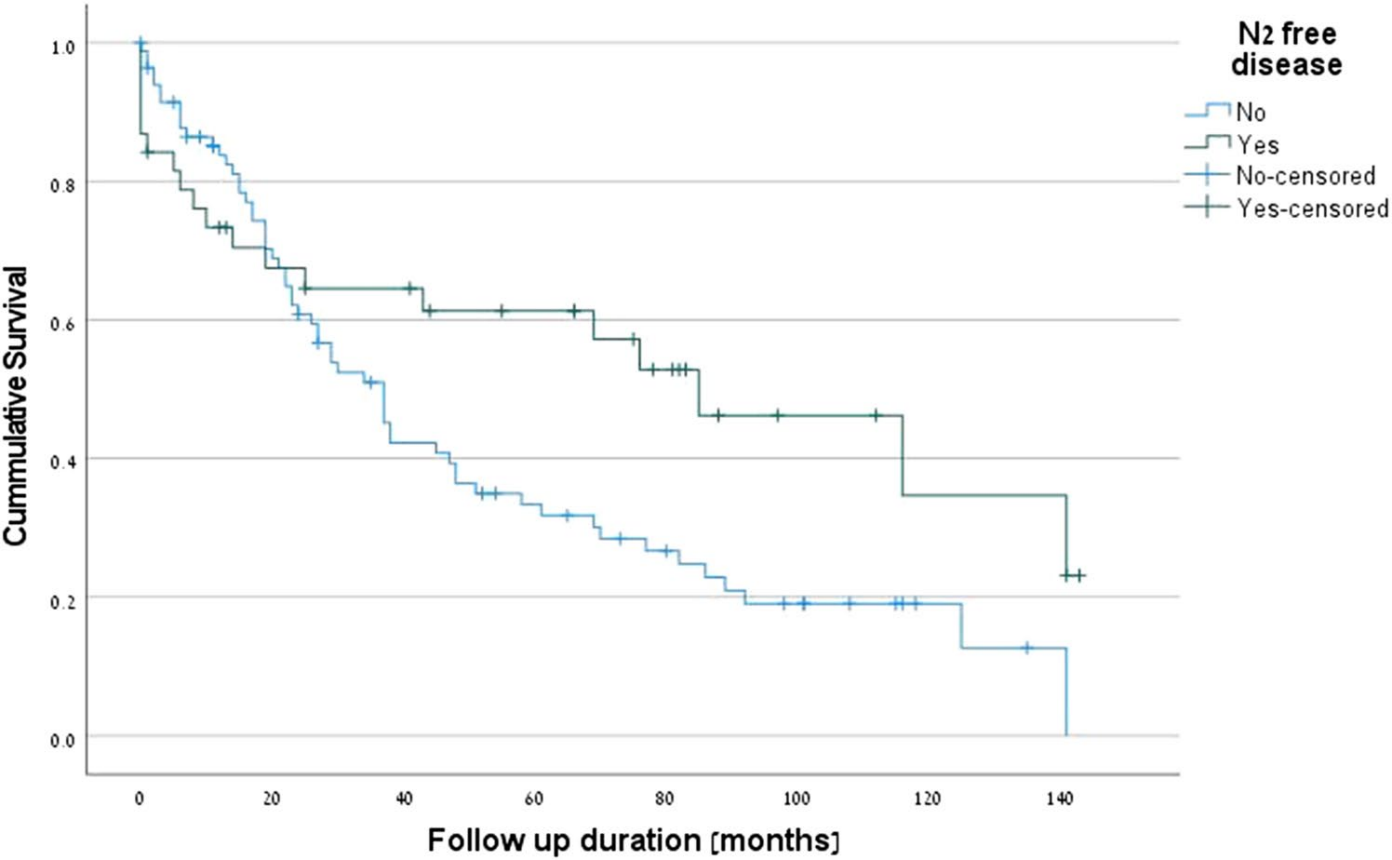
Kaplan–Meier survival curves for preoperative N2-free disease.

**Figure 4:**
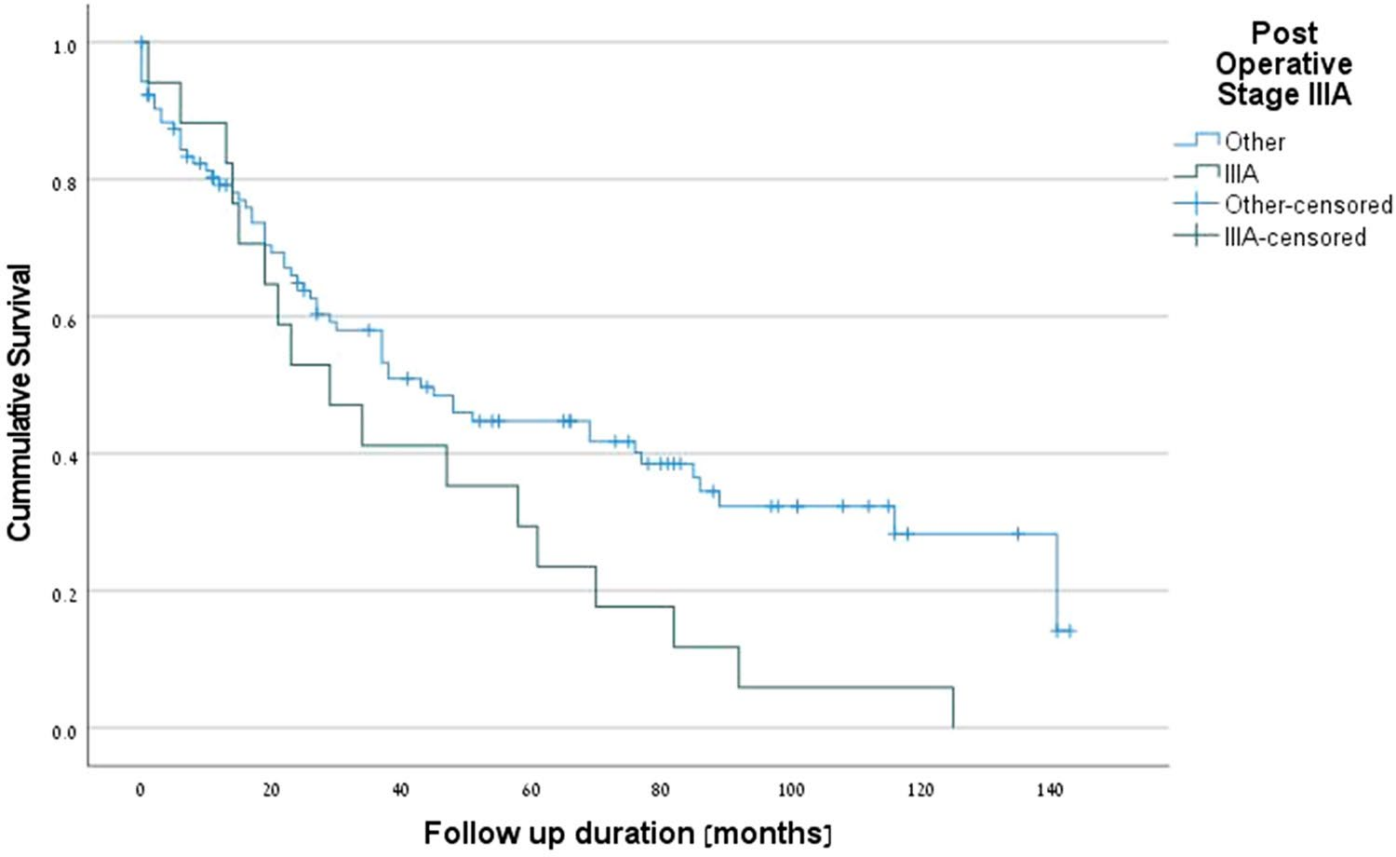
Kaplan–Meier survival curves for postoperative stage IIIA.

**Figure 5:**
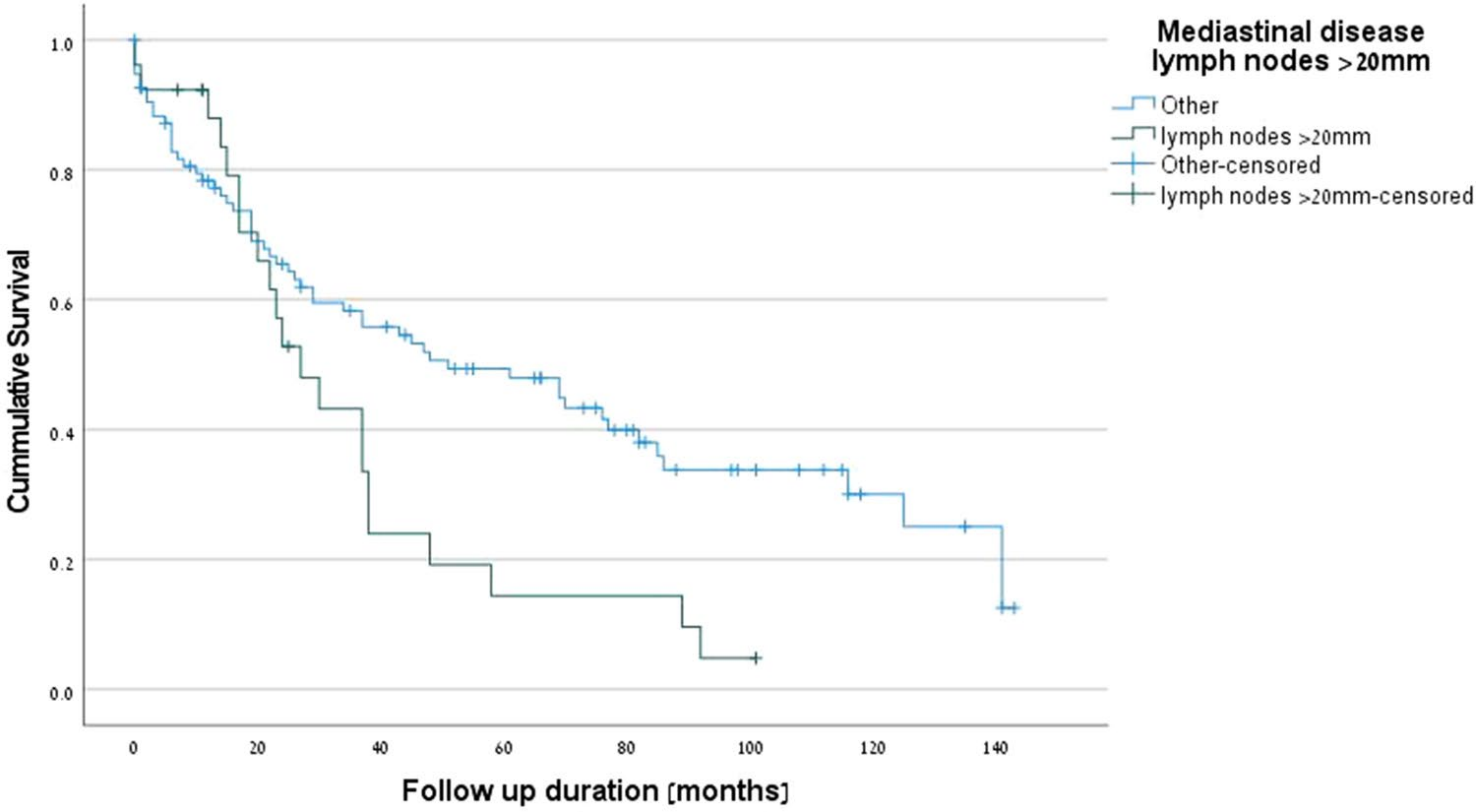
Kaplan–Meier survival curves for bulky mediastinal disease (lymph nodes > 20 mm).

The positive predictive factors for survival were PET-CT (p = 0.001) (Fig. 1), complete pathologic response (*p* = 0.001) (Fig. 2), R0 margins (*p* = 0.027), pre-neoadjuvant N2-free disease (*p* = 0.026) (Fig. 3), and intra-pericardial resections (*p* = 0.048). The negative predictive factors for survival were postoperative stage IIIA (p = 0.001) (Fig. 4), pre-neoadjuvant bulky mediastinal disease (lymph nodes > 20 mm) (p = 0.034) (Fig. 5), persistent postoperative N2 disease (p = 0.016), R1 resection (p = 0.027), and preoperative N2 multistation disease (p = 0.053). Follow-up was complete for 121 patients (97.6%), the overall 5-year survival rate was 47.9% (58 patients), and the survival to date (January 1, 2020) was 34.7% (43 patients).

## Discussion

Management of patients with locally advanced NSCLC remains one of the major challenges of thoracic oncology^8^. The treatment strategy is influenced by the disease stage, the patient's functional status, and the decisions reached by the interdisciplinary discussions at the institutional tumor board meetings. Resectable patients benefit from surgery when radical resection is achieved without major morbidity and mortality. Patients with locally advanced NSCLC undergoing surgery without preoperative oncologic treatment, chemotherapy, or chemoradiation, however, are at a greater risk of developing distant metastases or locally recurrent disease. There is a statistically confirmed survival benefit of neoadjuvant chemotherapy followed by surgery compared with surgery alone^9–13^, but the main concern with regard to surgery is the increased risk of postoperative morbidity and mortality^3,4,14,15^.

Our study aimed to identify predictors of postoperative outcome and survival of locally advanced non-small cell lung carcinoma (NSCLC) resections, including extended resections (extended pneumonectomies and others) after neoadjuvant chemotherapy or chemoradiation. Pneumonectomy remains associated with high and possibly unacceptable rates of perioperative morbidity and mortality, especially after induction therapy^16–19^. The recommendation of the American College of Chest Physicians (ACCP) is to avoid performing pneumonectomy after neoadjuvant chemoradiotherapy^20^. For example, Thomas et al.^16^ found that independent predictors of mortality in patients who received induction therapy followed by pneumonectomy were age greater than 65 years, male sex, ASA score of three or greater, and right laterality of the procedure. In contrast, Mansour et al.^21^ and Refai et al.^22^ did not find any significant differences in terms of early or late morbidity and mortality when comparing patients undergoing pneumonectomy after induction chemoradiotherapy with those who had no form of induction treatment. In our previous study, we also concluded that pneumonectomy could safely be performed after induction therapy with low (0%) early and late 2.4% (1 patient) mortality rates (17 pneumonectomies from 41 studied patients (41.7%))^23^. In our recent study, the rate of pneumonectomies performed was 49.2% (61 patients), most of them conducted after neoadjuvant chemoradiation (45 patients (36.3%)). Twenty-three patients (18.7%) underwent extended pneumonectomies (completion, extrapleural, intra-pericardial resections).

Detterbeck et al.^24^ reported 5-year survival rates of 19%/24% for clinical/pathological stage IIIA NSCLC (TNM 7) and 7%/9% for clinical/pathologic stage IIIB NSCLC (TNM 7). Andre et al.^25^ reported 5-year survival rates of 35% versus 5% if ipsilateral single mediastinal node versus multiple mediastinal station lymph node involvement was detected. The PACIFIC study was the first randomized trial that demonstrated a benefit in progression-free survival after simultaneous chemoradiotherapy with immunotherapy by durvalumab (PDL-1 inhibitor), with significantly prolonged overall survival compared to placebo (*p* = 0.0025) in patients with unresectable stage III NSCLC^2^. The limitations of the PACIFIC study were the exact staging and restaging methods and the high local recurrence rate^26^. The SAKK 16/14 study is a recent trial that demonstrated a new standard of care for 55 studied patients with resectable stage IIIA (N2) NSCLC treated with neoadjuvant chemotherapy with cisplatin and docetaxel followed by surgery and perioperative durvalumab, with 73% 1-year event-free survival, 2% perioperative mortality (one patient with fatal bronchopulmonary bleeding), 93% postoperative R0 resection (51 patients) and 18% complete pathologic response (10 patients)^27^. Five-year survival rates after neoadjuvant chemotherapy or chemoradiation have been reported to range from 21 to 41%^11–13,28^. Van Meerbeck et al.^28^ reported that only complete resection has a positive impact on survival and that the results of incomplete resection are compatible with the results of patients treated solely with radiotherapy (with a 5-year survival of 15.7%). The overall 5-year survival rate reported in our study was 47.9%; complete pathologic response was achieved in 35.5%, and complete resection was achieved in 92.7% (with only one patient (0.8%) exhibiting local recurrence). Most of 44 patients with CPR underwent neoadjuvant chemoradiation (35 patients (29.3%)), and only 9 patients underwent neoadjuvant chemotherapy only (6.2%). Koshy et al.^11^ and Kim et al.^29^ reported 5-year and 61% 5-year survival rates, respectively, in patients achieving a pathologic complete nodal response.

Martin et al.^30^ concluded that pulmonary resection after neoadjuvant chemotherapy is associated with acceptable morbidity and mortality, with right pneumonectomy (*p* < 0.002), blood loss (*p* < 0.001), and forced expiratory volume in one second (*p* < 0.001) being predictive risk factors for postoperative complications. Weder et al.^31^ demonstrated low perioperative mortality (3%) and acceptable major morbidity (13%) rates, and Kim et al.^29^ showed a 3% rate of early 30-day mortality and an 8% rate of 90-day mortality in patients who underwent pulmonary resection after neoadjuvant chemoradiation. The recent Check Mate 816 investigation was a randomized phase 3 study of neoadjuvant chemotherapy by nivolumab (NIVO) + platinum chemotherapy vs. only platinum chemotherapy in resectable NSCLC^32^. Lobectomy was performed in 77% vs. 61% and pneumonectomy in 17% vs. 25% of 149 and 135 patients in the NIVO + chemotherapy and chemotherapy only arms, respectively, with 0 vs. 3 deaths, 83% vs. 78% R0 resection, and 10% vs. 74% residual viable tumor cells in the primary tumor bed in the two groups, respectively^32^. In our study, we reported the rate of postoperative complications in 49.2% of the patients, mainly atrial fibrillation (15.9%) and pneumonia (13.7%), empyema (10.3%), and early bronchopleural fistula (7.3%). The early in-hospital mortality rate was 6.5%, and the 6-month mortality rate was 5.6%.

We statistically evaluated different parameters of patients with locally advanced potentially resectable disease to analyze the predictive factors that potentially influence postoperative outcome and found that most of them were nonsignificant. We determined that pre-neoadjuvant bulky mediastinal disease (lymph nodes > 20 mm) or central extension of the tumors to the proximal airways (p = 0.034), persistent postoperative N2 disease (p = 0.016), positive surgical margins (p = 0.027), preoperative N2 multistation disease (p = 0.053) and postoperative stage IIIA (p = 0.001) were negative predictive factors that influenced patients’ postoperative outcome and survival. We also found that complete pathologic response (*p* = 0.001), negative surgical margins (*p* = 0.027), pre-neoadjuvant N2-free disease (*p* = 0.026), and intra-pericardial or extended major pulmonary resections (*p* = 0.048) were positive predictive factors that influenced postoperative outcome and survival. According to the results of our study, we reassessed our surgical policy to perform more lung-sparing surgeries after neoadjuvant chemotherapy or chemoradiation instead of pneumonectomies.

Mc Elnay et al.^33^ posed the key question of whether surgery should be considered as part of multimodality treatment for patients with resectable lung cancer and ipsilateral mediastinal nodal disease. The authors concluded that there were no significant differences in overall survival in patients randomized to surgery as part of bimodality (chemotherapy + surgery) or trimodality (chemoradiation + surgery) treatment^33^. Other studies that reviewed randomized evidence of radiochemotherapy versus surgery within multimodality treatment in stage III NSCLC found no significantly different overall survival in patients with locally advanced NSCLC after induction treatment and surgery compared with those receiving definitive radiochemotherapy^34,35^. Arguments in favor of surgery in patients with resectable disease refer to large residual necrotic tumors, which are difficult to control with radiotherapy and may lead to the formation of a lung abscess or to multiple nodules in the same lobe. The combination of preoperative concurrent chemotherapy and radiotherapy followed by surgery should be considered where local control is especially important for quality of life, such as with invasion of the brachial plexus in superior sulcus tumors (Pancoast tumors) or central tumors without mediastinal nodal disease. A pooled analysis of three SAKK trials (SAKK 16/96, 16/00 AND 16/01) examined long-term results for 368 operable stage III NSCLC patients divided into bimodal (neoadjuvant chemotherapy + surgery, 205 patients) and trimodal (neoadjuvant chemoradiation + surgery, 163 patients) and reported 56 (33%) vs. 42 (31%) pneumonectomies in each group, with 7% vs. 2% postoperative mortality (36). The authors demonstrated 5- and 10-year survival rates of 38% and 28% for stage IIIA NSCLC and 36% and 24% for stage IIIB NSCLC in the two groups, respectively. They reported 69.2% (119 patients) and 87.2% (117 patients) R0 resection and 14.5% (25 patients) and 16.4% (22 patients) complete pathologic response in the two groups, respectively, and showed that R0 resection and CPR together with younger age were associated with improved survival (p = 0.043, p < 0.001, p = 0.009, respectively)^36^.

Looking back upon more than 20 years of performing major pulmonary resections after neoadjuvant chemotherapy and chemoradiation, we must admit that although our results are compatible with the abovementioned studies, not all of the outcomes were good, and some were inarguably bad**.** It is our impression that with the selection of good surgical candidates and after achieving complete pathologic response, negative surgical margins and R0 radical resections, major pulmonary resections can be considered an acceptable treatment option in certain patients with stage III potentially resectable NSCLC.

Our study has several potential limitations. This was a retrospective nonrandomized study, which is subject to selection bias; the data regarding pneumonectomy rates in our investigation were significantly higher than those in other reports, and we are also aware of the need for more scrupulous preoperative mediastinal staging after neoadjuvant chemotherapy or chemoradiation to reduce the incidence of postoperative N2 disease.

## Conclusions

Based upon the results of the current study and our 20 years of experience operating on post-neoadjuvant patients, we recommend avoiding resections in patients with bulky mediastinal disease and avoiding pneumonectomy in patients with centrally located tumors involving the carina and esophagus before administering neoadjuvant chemotherapy or chemoradiation, and we prefer lung-sparing resections. Moreover, we strongly recommend surgery for patients with locally advanced potentially resectable NSCLC without the abovementioned contraindications, including pneumonectomies, if such procedures are performed in highly experienced thoracic oncology surgery departments and with low mortality and acceptable morbidity.

## Data Availability

The datasets used and/or analyzed during the current study are available from the corresponding author on reasonable request.
